# Epigenetic control of variation and stochasticity in metabolic disease

**DOI:** 10.1016/j.molmet.2018.05.010

**Published:** 2018-05-18

**Authors:** Ilaria Panzeri, John Andrew Pospisilik

**Affiliations:** Max Planck Institute of Immunobiology and Epigenetics, Stuebeweg 51, 79108, Freiburg, Germany

**Keywords:** Phenotypic variation, Inheritance, Metabolic diseases, Epigenetics

## Abstract

**Background:**

The alarming rise of obesity and its associated comorbidities represents a medical burden and a major global health and economic issue. Understanding etiological mechanisms underpinning susceptibility and therapeutic response is of primary importance. Obesity, diabetes, and metabolic diseases are complex trait disorders with only partial genetic heritability, indicating important roles for environmental programing and epigenetic effects.

**Scope of the review:**

We will highlight some of the reasons for the scarce predictability of metabolic diseases. We will outline how genetic variants generate phenotypic variation in disease susceptibility across populations. We will then focus on recent conclusions about epigenetic mechanisms playing a fundamental role in increasing variability and subsequently disease triggering.

**Major conclusions:**

Currently, we are unable to predict or mechanistically define how “missing heritability” drives disease. Unravelling this black box of regulatory processes will allow us to move towards a truly personalized and precision medicine.

## 1 Introduction – phenotypic variation

Understanding how phenotypic variation arises and the extent to which it is reproducibly plastic are fundamental biological questions. To understand phenotypic variation is to understand mechanisms that shape and drive differential disease susceptibility. Variation is the template for natural selection and therefore its investigation is also a means towards understanding speciation. Indirectly, this question has become *the* overarching goal of life science research over the last decades [1].

**Figure 1:**
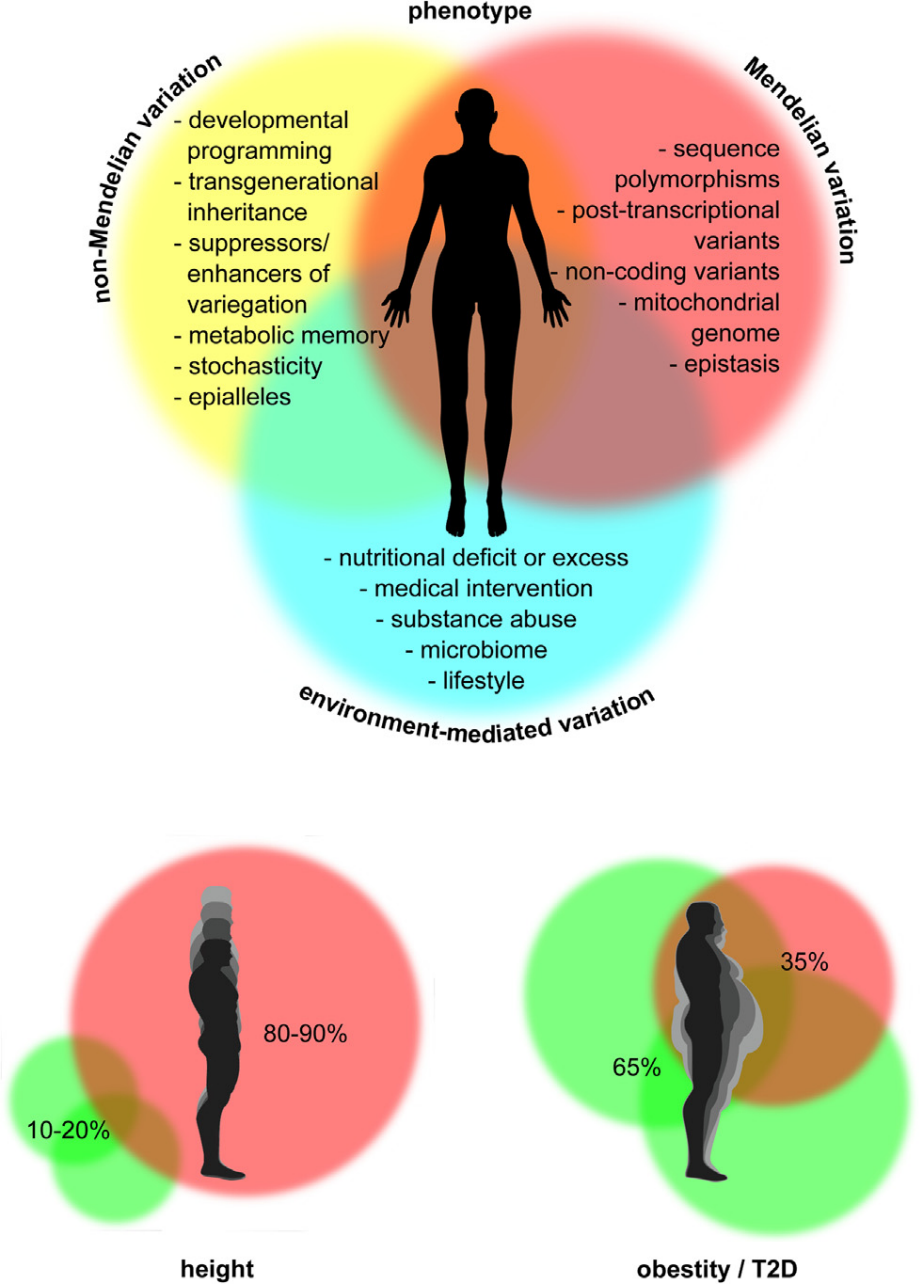
**Modes of variation impacting on phenotype**. Mendelian variation, non-Mendelian variation and the environment concur to modify the phenotypic outcome of an individual, often intermingling into each-other. The contribution of Mendelian variation to height determination is estimated around 80–90%, whereas for obesity/T2D estimates are lower (∼35% heritability).

**Figure 2:**
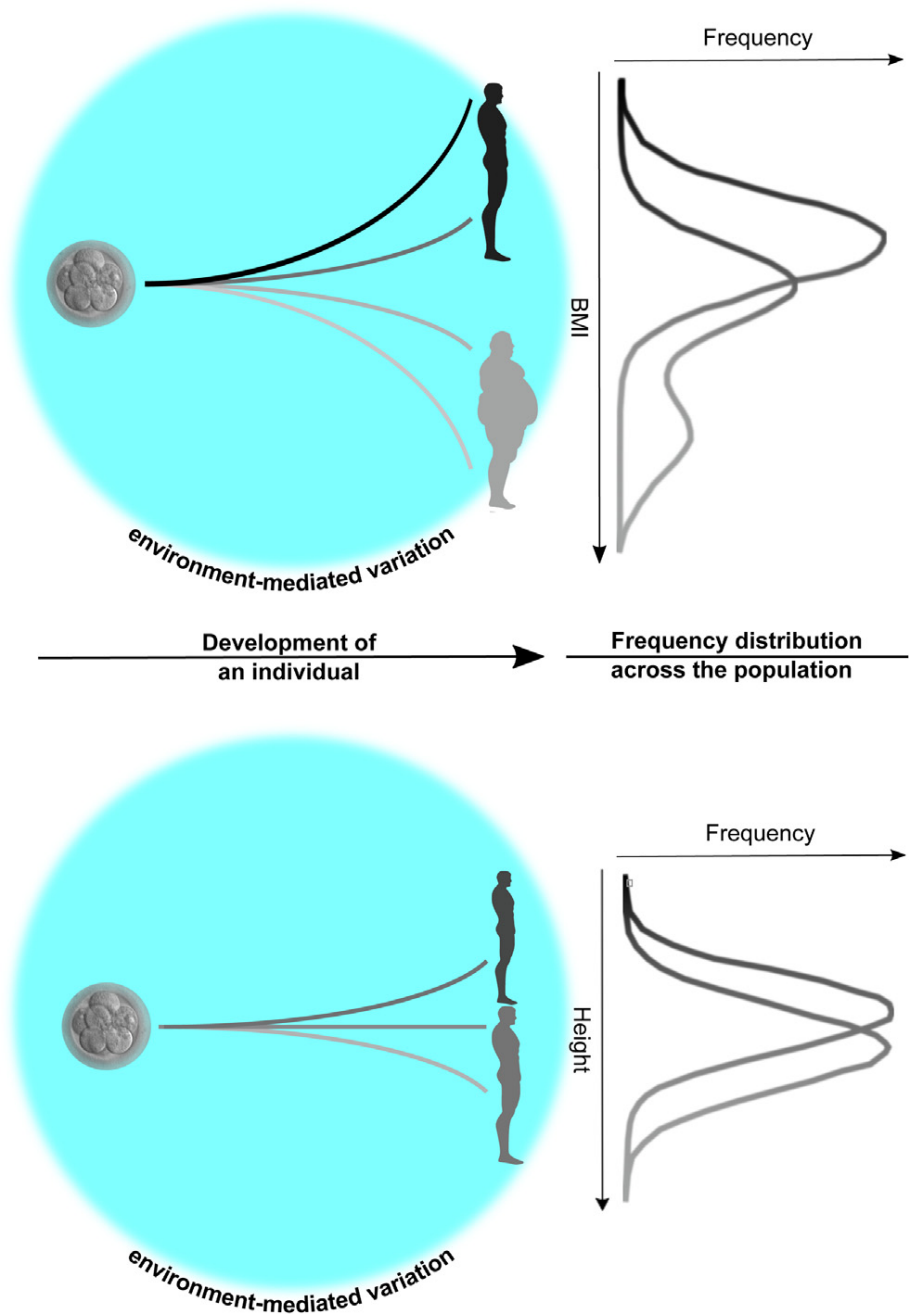
**Distributions of phenotypic variation.** Non-Mendelian phenotypic variation acts during development and can be described either as a discrete, combined or as a continuous distribution. In the first scenario the traits subject to the variation are termed ‘polyphenic’ (here exemplified by BMI, whereas height is exemplifying continuously varying traits).

Simply put, an organism's complex trait composition is the product of genetic, epigenetic, and environmental inputs and their cumulative interactions through development (Figure 1). The genetic mechanisms that control complex traits are ‘Mendelian’ in their transmission pattern (with some exceptions). They and comprise DNA-sequence differences and are responsible for the bulk of phenotypic variation across the human population. Epigenetic contributors, by contrast, are ‘non-Mendelian’ and rely on mechanisms that bypass DNA sequence to impart phenotype. Non-Mendelian or epigenetically-driven phenotypic variation (EPV) can be thought of, anecdotally, as variability that occurs in inbred populations, and can be classified according to distribution as either continuous (e.g. height) or discrete (e.g. flower color), or a combination (eusocial insect morphs). The latter examples are termed ‘polyphenisms’, scenarios in which the same genotype yields distinct phenotypes within a population, with no intermediates (e.g. worker and soldier ant). To the best of our knowledge, both continuous EPV and polyphenisms result predominantly from developmental responses to the environment (Figure 2), a broader phenomenon termed ‘phenotypic plasticity’.

**Figure 3:**
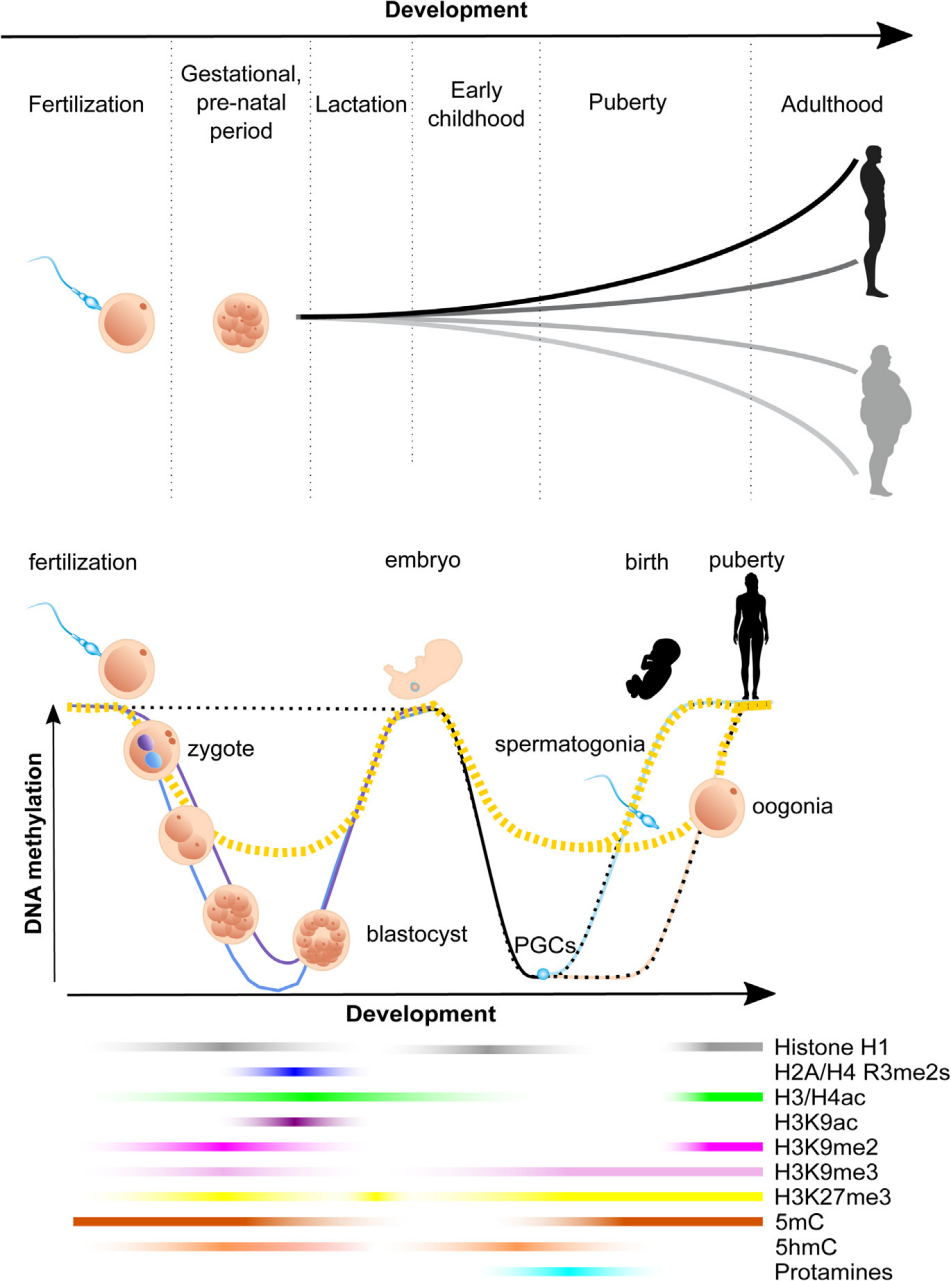
**Windows of sensitivity during development.** From the moment of conception throughout adulthood, the environment modulates and defines the phenotypic outcome of an individual, particularly impacting highly sensitive windows. These windows of sensitivity parallel the major morphological and epigenetic sub-divisions of development. *Blue line*: paternally derived alleles; *light blue line*: re-establishment of DNA methylation at paternally derived alleles after gametogenesis; *purple line*: maternally derived alleles; *pink line*: re-establishment of DNA methylation at maternally derived alleles after gametogenesis; *dark line*: epigenetic reprogramming in Primordial Germ Cells (PGCs); *dotted dark line*: alleles that are protected from epigenetic reprogramming at fertilization (imprinted genes, repetitive sequences, heterocromatin near centromeres); *dotted yellow line*: Endogenous RetroViruses (ERVs) and metastable epialleles can fully or partially escape epigenetic reprogramming. Epigenetic inheritance through generations can occur from incomplete epigenetic reprogramming (Cantone and Fisher, 2013).

**Figure 4:**
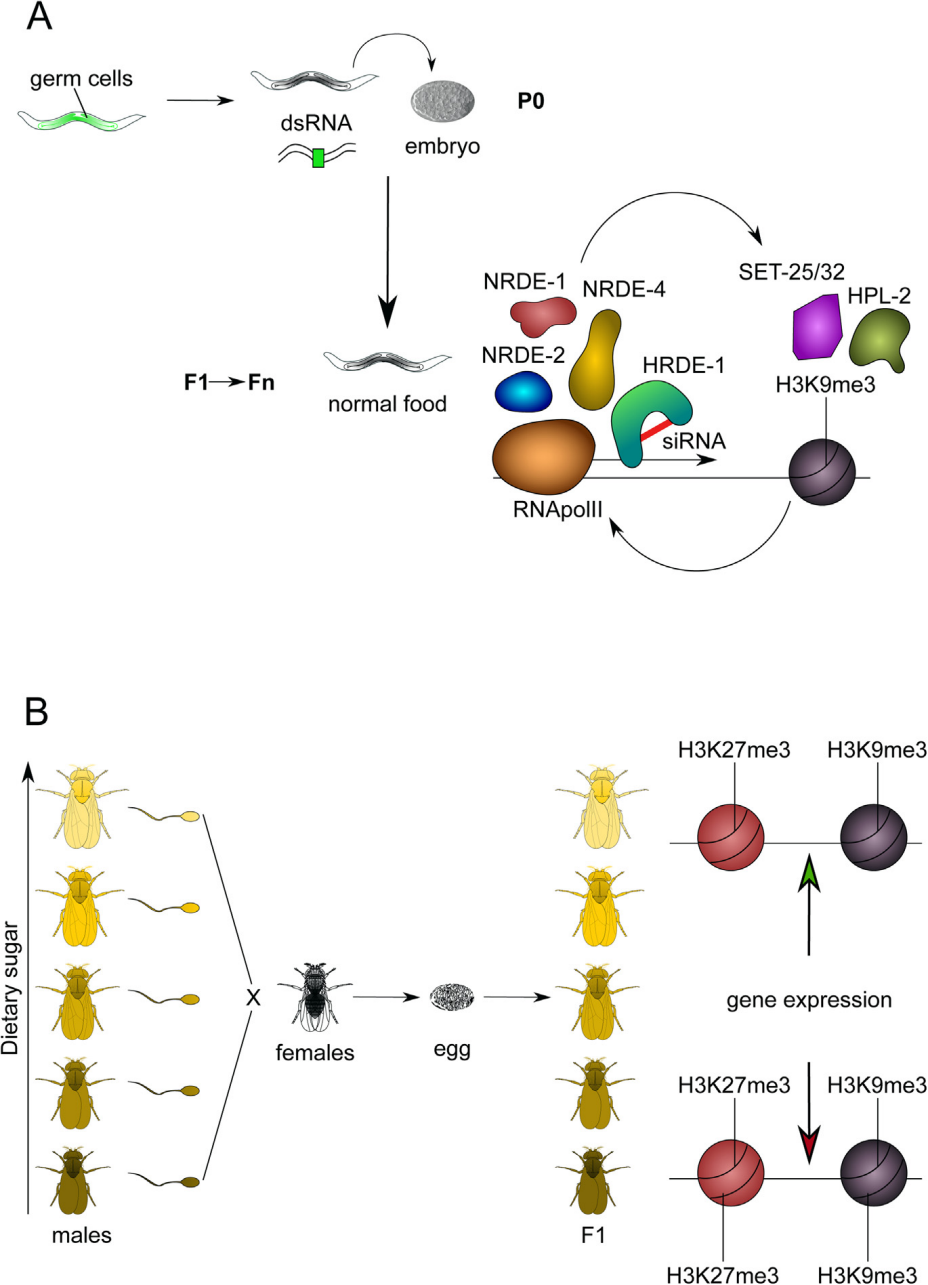
**Intergenerational epigenetic inheritance.** (A) In *C. elegans*, dsRNAs in food can silence an endogenous reporter. This repression is maintained for more than 20 generations in absence of the initial silencer. This maintenance of silencing requires nuclear RNAi factors and chromatin modifying proteins (Ashe et al., 2012). (B) In *D. melanogaster* (below), adult fed an obesogenic high-sugar exhibit a typical U-shaped obesity response with low- and high-sugar sired individuals showing exaggerated triglycerides and the latter also characterized by increased body weight. This intergenerational metabolic reprogramming results from global alterations in chromatin state integrity, particularly from reduced H3K27me3 and H3K9me3 domains (Öst, Lempradl et al., 2014).

Studies suggest that phenotypic plasticity is triggered preferentially during critical windows of high sensitivity including embryonic development and growth phases (Figure 3). More recently, the preconceptual germlines of the parents, and even ancestral germlines have been highlighted as additionally relevant ‘intergenerational’ windows of sensitivity for triggering plasticity (Figure 4).

## 2 Mendelian phenotypic variation – the template for plasticity

While single genetic variants alone are capable of causing disease (e.g. ‘MODY’ diabetes mutations), complex traits such as glycemia represent the physiological output of many contributing genetic loci and are therefore typically normal or log-normal distributed. Experimentally, mapping genetic architectures that drive complex traits is challenging. Quantitative physiological traits typically represent the integrated output of numerous cellular, tissue-specific, and inter-tissue effects. Blood glucose for example integrates organismal behaviors [exercise, depression etc.], multiple tissue responses [liver, muscle, adipose, etc.], and different hormonal axes [cortisol, glucagon, insulin, etc.], all of which comprise many gene interaction networks that are dynamic again to the environment. Most disease traits, in turn, are composites of *many* complex traits, exacerbating the challenge. Further, at the genic level, dozens, if not hundreds, of relevant alleles can exist in the population. A recent survey of coding sequence variation across 60,706 individuals identified 7.9% of high-confidence regions as multiallelic, i.e. contained multiple distinct sequence variants [2]. LDLR, the gene coding for the receptor for low-density lipoprotein (LDL) cholesterol [3], contained over 500 different missense mutations and 200 small insertions and deletions across the population. The challenges and complexities of quantitative trait genetics in the human population can be found here [4].

While DNA-sequence differences are the major contributors to population-level variation and they are not trivial to map, they represent the template upon which epigenetic mechanisms can act. Briefly therefore, sequence variations themselves include:

### 2.1 A range of variant types

Phenotypic variation can result both from gain- and loss-of-function mutations. Both can arise from DNA sequence insertions, deletions, and copy number variations (CNVs), simple mechanisms that affect gene dosage. Large deletions and duplications have been reported in many genes related to metabolic syndrome, including the *LDLR*, the lipoprotein lipase (*LPL*) and lamin B (*LMNB2*) genes [5].

### 2.2 Variants in non-protein-coding genes

The ∼98.5% of our genome is non-protein-coding: it is pervasively transcribed, and its transcripts can support regulatory function [6], [7]. Among the best functionally characterized non-coding RNAs (ncRNAs) arising from these sequences are microRNAs (miRNAs). These transcripts have been shown to be involved in conferring to cells [8], as well as in invertebrates and insects [9], biological robustness [10]: the capacity to maintain phenotype in spite of internal or external perturbations [11]. Interestingly, lncRNAs have been recently shown to exhibit higher natural expression variability among individuals compared to protein-coding genes [12].

### 2.3 Epistasis and background effects

Because of the extreme interconnectivity of cell regulatory networks, even at the cellular level, predicting the impact of a sequence variant is difficult as the resultant variation acts in the context of all other variants and their potential additive, synergistic and antagonistic interactions. This phenomenon is known as epistasis [13]. Recently, mouse chromosome substitution strains (CSSs) were used as a model to map quantitative trait loci (QTLs) on a fixed genetic background. Analysis of CSSs indicate that epistatic interactions control the majority of the heritable variation in both fasting plasma glucose levels and hepatic gene expression [14]. Along similar lines, any individual mutation or variant is susceptible to epistatic interactions that are individual-dependent, or in the case of inbred models, background-dependent as shown in different mouse lines [15] and, even more striking, within the same C57BL6 strain [16].

### 2.4 Non-coding variants

The last decade has seen quantitative trait genetics applying two major technical approaches to understanding variation. Genome-wide association study (GWAS; now replaced by exon and subsequently whole genome sequencing), which searches for association between *disease traits* and DNA-sequence ‘linkage’ blocks, have suggested that ∼88% of the disease-associated common genetic variants fall into non-coding areas of the genome [17]. Expression Quantitative Trait Locus (eQTLs) mapping, searches instead for associations between DNA-sequence linkage blocks and *gene expression*. In contrast to *cis*-eQTLs that affect gene expression of nearby genes, *trans*-eQTLs can impact genes located far on the same chromosomes or even on different chromosomes thanks to higher-order modular organization of gene regulation [18].

### 2.5 Post-transcriptional modification

A wide range of mechanisms contribute to functional output of a locus beyond eQTL mappable *cis-* and *trans-*regulation. Included are alternative splicing [19], polyadenylation, cleavage and processing of polypeptides, differential binding and function of subunits as well as post-translational modifications (PTMs). All these layers of regulation have a DNA-sequence basis, and therefore can be subject to DNA-sequence variation. Such effects are not rare. Genetic variants that affect RNA processing are as abundant as those that affect transcription, and aberrant effects can lead to disease [20]. Additional, somewhat arbitrary examples include the identification of splicing-associated SNVs related to autoimmune type 1 diabetes and rheumatoid arthritis [21] and of SNVs altering conserved polyadenylation signals in the insulin gene causally linked to neonatal diabetes [22].

### 2.6 Mitochondrial genome and the mitonuclear interactions

Many lines of evidence now implicate mutations affecting the mitochondrial genome in metabolic disease. The impact of these mutations affects at least 1 out of 5000 people [23]. Pathogenic alleles can be traced in more than 1 out of 200 live births and occur *de novo* in at least every 1000 births [24]. Though mitochondrial DNA (mtDNA) represents less than 1% of the total cellular DNA, mitochondria play a central role in energy metabolism, cell fate control and apoptosis [25]. Recently epistasis and ‘mitonuclear interactions’ between mtDNA and nuclear DNA have also been identified [26], [27]. Particularly, mtDNA variants, through their effects on metabolism, can induce epigenetic alterations within the nuclear genome and thus influence stress and cancer [28].

### 2.7 Gene–environment interaction

Gene expression is constantly modulated by external and internal cellular signaling, and thus by cellular or organismal ‘environment’. By extension, organismal phenotypic variation is responsive to environmental factors, and genetic variation can be revealed, at times, only under certain environments (gene–environment interaction, Figure 1). The term ‘environment’ includes multiple layers of external variables that influence a trait. In case of glycemia or adiposity, these include diet, exercise, and stress, and are essentially countless. They can also include internal regulatable factors such as microbiome composition. In this case the influence is reciprocal: our nutrition and genetics shape the composition of microbiota, and thus our own environments [29], [30].

In budding yeast, accurate prediction of individual growth rates is greatly improved knowing not only genome sequence but also environment [31]. In humans, the *FTO* locus not only associates extremely robustly with BMI [32], [33] but also it is a unique example of a locus that associates with *phenotypic variation in* BMI [34]. Interestingly, physical activity attenuates (by ∼30%) the influence of the *FTO* locus on body mass index (BMI). This study demonstrates that interactions between genes and lifestyle exists in human and importantly that a given genetic susceptibility is modifiable by lifestyle [35].

### 2.8 Summary

A complex architecture of genetic variation drives the majority of complex trait heterogeneity in the population. With each individual predicted to have ∼4 million genetic variants, deconvoluting this puzzle remains a tall task for quantitative geneticists [36]. That said, efforts are paying off with regular reports of novel genetic driver paradigms for human disease. With this ever more robust genetic understanding, biologists are now ready to mechanistically tackle the more subtle and elusive sources of variation, and in particular non-Mendelian, and environmentally driven interactions.

## 3 Non-Mendelian phenotypic variation

Since the 1980s we have known that ‘imprinted’ loci can escape the erasure of DNA-methylation control that occurs during gametogenesis and early embryogenesis, enabling robust parent-of-origin patterns of gene expression during development and across generations. More recently, ERVs and less-well characterized ‘metastable epialleles’ have been demonstrated to escape reprogramming [37]. These pioneering demonstrations provided molecular proof for the existence of at times robust, but non-Mendelian mechanisms that underpin gene expression effects and thus phenotype. Numerous examples now exist in nature (wasps, ants, and butterflies), from tracking spontaneous phenotypic mutations in the lab (A^vy^ and Kit^∗^ mice) and from experimental efforts across diverse model organisms (PEV in yeast and Drosophila, and Momme's in mice), that stark non-Mendelian mechanisms exist to reproducibly impact phenotype. While still a matter of debate, evidence from inbred models and human twin studies indicate non-heritable mechanisms contribute significantly to variation and disease.

### 3.1 Windows of sensitivity

What has been learned from the stark contrasting examples listed above (eusocial insects, PEV systems, and epimutant mice) is that a fraction of adult trait and disease etiology traces back to environmentally sensitive mechanisms and decisions made during development [38]. In the context of human disease, this notion was coined ‘developmental origins of health and disease’ (DOHaD), a hypothesis for which there is now ever-increasing evidence. Environmental variation or ‘stimuli’ occurring during critical windows of susceptibility can elicit lifelong alterations in an individual's phenotype [39]. Reviewing the literature on all the environmental stimuli that have been documented to drive phenotypic plasticity is beyond the scope of this article. What is clear from this literature though is that a series of developmental windows exist during which the organism is most susceptible, and that these windows differ for each environmental trigger – phenotype pair. These windows parallel the major morphological and epigenetic sub-divisions of development, suggesting hypotheses about the response mechanisms underlying the plasticity and stabilization (Figure 3).

Below we highlight a select few paradigms organized according to time-windows including preimplantation (totipotency, pluripotency), early and mid-gestation (formation of the germ layers and their subsequent differentiation), as well as late gestation and early life (growth, organ maturation, birth and transition to solid nutrient diet). Many unanswered questions remain about these models of plasticity and their relevance for human variation and disease. How many environmental variables are read out in variation of any given trait? How many windows of susceptibility exist for any given trait? How many environmental triggers are being integrated? Is there hierarchy amongst triggers? How reversible are programming effects? What are the windows of reversibility? How plastic is a developmental trajectory once established? Can diagnostics for plasticity be developed? And, most importantly, what are the organismal, cellular, and epigenetic mechanisms that generate and hardwire plasticity phenotypes.

#### 3.1.1 Preconception and intergenerational epigenetic inheritance

A recent surge of studies has highlighted the influence of *preconception* parental exposures on offspring phenotype. Examination of reciprocal two-cell embryo transfers between lean and obese female mice – an attempt to contrast plasticity effects from maternal germline vs maternal gestation environments – demonstrated that two-cell embryos from obese mothers exhibit impaired fetal and placental growth [40]. Interestingly, the behavioral reward system of the same offspring was also altered in adulthood, though it is unclear whether that phenotype is programmed into the 2-cell embryo or derives from the impaired placental effects [41].

In addition to a large literature on the role of maternal/gestational effects [42], a recent surge of effort has explored paternal effects. Whereas gestation includes many mechanistic windows of sensitivity, the oocyte-embedded maternal and sperm-embedded paternal effects transmit in a ‘singular’ developmental moment, conception. Focus on paternal effects have gained popularity in part because of their logical, but also experimental, simplicity. Males primarily transfer sperm, seminal fluid, and some behavioral effects during mating [43], [44]. Experimentally *in vitro* fertilization (IVF) can be leveraged to focus on information that is transmitted by sperm, making male gamete intergenerational effects a tractable experimental problem. Paternal dietary effects have been reported for a range insufficiency (caloric restriction, low protein, low-methyl donor) to excesses (high fat; HFD). Male rats maintained on HFD during adulthood sire daughters with mildly impaired glucose tolerance and abnormal pancreatic morphology [45]; in mice, this is true both for sons and daughters [46]. Similarly, males on low-protein diets father offspring with impaired glucose tolerance and blood pressure [47], decreased hepatic cholesterol, and increased expression of genes related to cholesterol biosynthesis [48]. Paternal stress and toxin-triggered insults have also been documented to induce long-term epigenetic effects on offspring [49], [50]. Importantly, these effects are relevant across phyla, with the most mechanistically clear examples generated in lower organisms. Indeed, a germline nuclear small RNA (RNAi) pathway triggered by a piRNA-dependent foreign RNA response has been shown to be able to maintain silencing in *Caenorhabditis* *elegans* through at least 20 generations, acting via chromatin factors (including Argonaute proteins, a HP1 ortholog and two SET proteins) [51]. In flies, as little as two days of acute obesogenic dietary sugar intervention can modify offspring phenotype via the male germline, with low- and high-sugar sired individuals showing exaggerated triglycerides and body weight of offspring increasing with paternal sugar. This intergenerational effect requires Polycomb- and heterochromatin plasticity, as gene expression increases upon paternal high sugar preferentially at heterochromatin-embedded genes in embryos [52], [53] (Figure 4). The best-known evidence of paternally-mediated effect on progeny in humans comes from the Overkalix cohort. Historical studies of fluctuation in food availability in this remote town in Northern Sweden show that limited food availability in grandparental adolescence or adulthood correlate with grandson type 2 diabetes (T2D), cardiovascular disease and longevity. Interestingly, inadequate diet in grandparental adolescence (9–13 years) correlates with decreased risk, whereas the same exposure in grandparents as young adults (18–22) was associated with increased risk [54], [55], [56].

What molecular mechanisms underlie germline transfer of such intriguing, intergenerational associations? In most paradigms, clear insight into the mechanism of transfer is lacking. The majority of attempts to explain heritability across generations have focused on ncRNAs as well as DNA methylation and histone modifications which have long been thought of as ultra-stable biochemically. Epigenetic marks can escape early reprogramming in the developing embryo [57]. This is particularly true for imprinted genes [58], [59] and some classes of retrotransposons [60], [61]. Isogenic mice carrying the *Agouti viable yellow (A*^*vy*^) allele are epigenetic mosaics for DNA methylation at the intracisternal A particle (IAP) retrotransposon controlling the expression of agouti protein [63]. As a result isogenic *A*^*vy*^ mice display a variety of coat colors from yellow to full agouti (pseudoagouti). Interestingly, the percentage of yellow pups decreases after exposure of the dam to a diet rich in methyl groups [64], [65]. Histone marks have also been reported to correlate with silencing at the *A*^*vy*^ locus in pseudoagouti mice [66]. Indeed, not all histones are cleared from the DNA during germline reprogramming. Approximately 1–2% of the haploid genome in mice and ∼4% in humans remain packaged into nucleosomes in sperm [67], [68]. Paternal diet can affect H3K27me3 status in sperm, demonstrating that sperm histone modifications may be sensitive to paternal nutritional cues [48]. Histone modifier dosage has been shown able to regulate intergenerational effects [69], [70]. Furthermore, even though the highly condensed sperm nucleus is transcriptionally inert, RNA populations have been detected in mature sperm [71], [72] and sperm-RNA in the zygote [73]. Particularly, the majority of small RNA isolated from cauda sperm from mice are tRNA fragments (tsRNAs) predominantly deriving from the 5′ ends of tRNAs [74], [75]. Evidence exists that these tRNA fragments as well as other small non-coding RNAs (sncRNAs, like piRNAs and miRNAs [76]) can be affected by low-protein diet [77], HFD [74], starvation [78], and stress [76], [79]. Injection experiments showed that these sncRNAs can in turn modulate the expression of targets in the offspring, transmit metabolic disorders [74], [76], [77] and behavioral alterations [76], [79].

#### 3.1.2 Gestation and early life

Central to the DOHaD hypothesis is the idea that developmental programming mechanisms have evolved to prepare the fetus for a life defined in part by the respective environmental stress. If the environment into which the child is born contrasts that of original stimulus, this inappropriate developmental programming is hypothesized to lead to disease. Epidemiological data from the 1944–1945 ‘Dutch hunger winter’ support this theory. Due to the ravages of the Second World War, the western part of the Netherlands suffered severe food shortage in this period, with as low as 400–800 kcal individual daily rations. A study performed in 1994 showed that starvation suffered by mothers in gestation in this period (corresponding to under-nutrition sensed *in utero* by the developing fetus) correlates with an increased risk for cardiovascular and metabolic disorders, including diabetes, in the offspring as well as increased susceptibility to pulmonary diseases, altered coagulation, and higher incidence of breast cancer. Indeed, subsequent compensatory growth during early life increased the risk of chronic disease in offspring [80]. In laboratory studies, when healthy rat and mouse dams are exposed to undernutrition during the last third of pregnancy, their offspring show ∼15% reduction in birth weight and experience early postnatal ‘catch-up growth’. These mice show progressive glucose intolerance, insulin secretory dysfunction, reduced muscle mass and obesity in adulthood [81], [82]. Similarly, maternal protein restriction by ∼40–50% of normal intake during gestation and lactation can impair insulin sensitivity and induce β-cell defects and hypertension in offspring [83], [84]. A contrast to the mismatch in developmental and post-natal environment highlighted by the Dutch hunger winter, babies born during the Leningrad siege appropriately developed a “thrifty phenotype” allowing them to survive the famine that spanned more than 800 days [85], [86].

Experimentally vetted cues sensed during gestation include not only nutritional deficit or excess but also endocrine-disrupting chemicals (EDCs), disease states, lifestyle, substance abuse, and medical interventions. Exposure to environmental chemicals can trigger future biases in adipogenesis and support development of obesity [87]. Mechanistically, such exposures have been shown able to bias stem cell fate, favoring differentiation of adipocytes at the expense of other cell fates (including bone), as well as influencing mature adipocyte biology. Such pro-adipogenic biases correlate with childhood predisposition to obesity [88]. Interestingly, maternal behavior during pregnancy or early childhood before weaning has been shown to impact offspring behavior and cognitive development. Stress during pregnancy is a well-recognized cue that impacts offspring in animal models. Also in humans, children from stressed mothers are more likely to develop anxiety or depression, Attention Deficit Hyperactivity Disorder (ADHD), and slow learning ability. Particularly, if maternal anxiety levels are in the top 15% of the general population, the risk for children increases by about 5–10% [89], [90]. Similar observations have been made for gestational diabetes and increased offspring birth weight, adiposity, and risk for obesity and diabetes [91]. Interestingly, studies from the Pima population indicate that this is not only a matter of shared genetics: offspring born from the same women before and after having developed diabetes are consistently different in terms of body weight, the second being consistently heavier [92]. Indeed, it is now clear that exposure to certain conditions in the womb can lead to epigenetic changes that do not involve alteration of the DNA sequence (non-Mendelian inheritance, further discussed in Chapter 2.3).

While birth, or the ‘intrapartum’ period, has been considered too short to enable lasting epigenetic effects, recent studies have highlighted a wide range of potential birth-associated health consequences [93]. Particularly, oxytocin or prostaglandin treatment and cesarean section have been linked to increased rates of autism spectrum disorder (ASD), ADHD, asthma, eczema, T1D, infant bronchiolitis, multiple sclerosis, and obesity [68]. Postnatal overnutrition induced by litter size reduction in animal models at birth can directly promote obesity, insulin resistance and glucose intolerance [94]. Maternal behavior during the early suckling period can influence offspring robustness through to adult life, likely through sustained alteration in DNA methylation and histone acetylation at the glucocorticoid receptor (GR) promoter [95]. While breastfeeding protects against later obesity in humans [96], animal data suggest that milk composition can program offspring with higher risk of obesity and metabolic diseases [97], [98]. For further examples on the developmental origin of metabolic diseases and particularly obesity, we suggest the following review [99].

Due in part to their reproducibility and maturity as experimental paradigms, gestational and early-life models are probably the best described with regards to phenotype and disease etiology. Still a key challenge in the field is to understand the physiopathological influence of the environment during development. Studies in animal models showed that many of the prenatal insults share common involvement of hormones, particularly insulin, leptin, and androgens, in both sensing and transmitting environmental information. Nonetheless, also other metabolic factors such as amino and fatty acids, oxidative stress and low-grade inflammation are involved [100]. Gestational obesity increases oxidative stress and inflammation related genes in the placenta [101] and in the skeletal muscle of the developing fetus [102] and HFD increases the expression of enzymes involved in oxidative stress in mice [103]. Indeed, in response to a reduction in energy supply, mitochondria are activated in the fetus to satisfy the cellular need for energy [104]. Activated mitochondria produce reactive oxygen species (ROS) and undergo oxidative stress, which can have detrimental effects on cells with high energy requirements, such as pancreatic β cells [105]. Maternal supplementation with antioxidants reduces this oxidative stress and prevents adiposity in rats fed with Western diet [106]. Epigenetic modifications are clearly key mediators in these processes as essentially all known epigenetic modifiers rely on intermediary metabolites such as S-adenosyl-methionine (SAM), Acteyl-Coenzyme-A (CoA), α-ketoglutarate, and NAD^+^ to exert their function [107]. Impaired glucose metabolism in women during pregnancy is associated with an altered DNA methylation at the leptin genes in both maternal and feto-placental compartments, with increased risk of developing adult obesity and type 2 diabetes in the offspring [108]. Changes in DNA methylation and histone acetylation in the promoter region of *Pdx1*, a transcription factor critical for β cell function and development, are associated with reduced pancreatic β cell mass and pre-diabetic state following intrauterine growth restriction (IUGR) in rats [109].

#### 3.1.3 Plasticity in the adult – residual programming potential & metabolic memory

Developmental time-windows for programming aside, evidence also supports residual plasticity in adulthood. Such effects, as well as the innumerable environmental differences between individuals even in controlled study situations remain one of the biggest hurdles in quantifying the sources of non-Mendelian variation.

Vascular complications related to type 1 and type 2 diabetes represent the primary drivers of morbidity and mortality in the diabetic population [110]. Intensive glycemic control seems to reduce development and progression of these complications. The Diabetes Control and Complications Trial (DCCT), and the follow-up Epidemiology of Diabetes Intervention and Complications (EDIC) study coined the term ‘metabolic memory’ to describe the long-term vascular benefits that can be observed at least 10 years after a period of intensive glycemic control [111], [112]. The United Kingdom Prospective Diabetes Study (UKPDS) [113], [114] described this as a ‘legacy effect’.

At the cellular level, transient exposure to high glucose induces persistent upregulation of extracellular matrix proteins, including fibronectin and type IV collagen in endothelial cells [115]. These findings are in line with the notion that endothelial dysfunction drives cardiovascular disease in diabetes [116], [117]. The proposed underlying mechanisms are complex and are hypothesized to involve both cellular and epigenetic mechanisms. In particular, modification of mitochondrial proteins via non-enzymatic glycation end products (AGE), oxidative stress and low grade inflammation appear at least indirectly to contribute to the aforementioned endothelial dysfunction, vascular cell growth, macrophage infiltration, fibrosis, inflammation, and general organ dysfunction [118]. To date though, clear mechanisms of memory, as opposed to self-exacerbating cellular dysfunction, remain scarce.

### 3.2 Mechanisms of non-Mendelian variation

While much literature exists describing the pathophysiology that arises in non-Mendelian variation paradigms, the discrete molecular mechanisms that initiate, buffer, and threshold such effects have been elusive.

#### 3.2.1 Environment ‘sensitive’ chromatin networks

The epigenetic players are key factors in modulating the expression of genes in response to the environment. Position Effect Variegation (PEV) systems in yeast and *Drosophila* have proven to be elegant systems not only to screen for novel epigenetic regulators but also to highlight the molecular pathways that underpin interactions between environment and chromatin control. PEV was first described in *Drosophila* upon observation that an X-ray induced mutation resulted in flies with an eye-color phenotype characterized by highly variable patterning both red and white pigmented cells [119]. Subsequent examination showed an inversion or rearrangement in the polytene chromosomes, with one breakpoint at the pericentric heterochromatin and one adjacent to the *white* gene. Since the isolation of this initial strain, a large number of different PEV rearrangements have been described, generated by ethane methyl sulphonate (EMS), X-ray treatment, or remobilization of P elements, a DNA transposon [120]. These fly lines have been used to screen for dominant second site mutations that are able to either suppress (‘Suppressors of variegation’ or Su(var)s) or enhance (‘Enhancers of variegation’ or E(var)s) the PEV effect. The first dominant modifier mutations for *white* variegation were described in the 1950s [121]; now overall, ∼140 Su(var) and ∼230 E(var) distinct mutants have been identified, with around 30 mapped to gene identity [120]. Notably, many of these have ultimately proven to be direct biochemical mediators of silent chromatin deposition or removal. Importantly, numerous clear examples exist that PEV systems (and by extension the gene networks that regulate chromatin silencing) can sense physiologically relevant changes in environment. PEV can indeed be modified by a plethora of factors, including temperature [122] and heat shock [123], food richness, or abundance [124], [125]. *Drosophila* has also been used as a model to study how paternal diet defines offspring metabolic state. Recently, we showed that paternal sugar consumption act as physiological suppressor of variegation, inducing de-silencing of spatially defined chromatin domains that can be traced both in sperm and in embryos. Mechanistically, full paternal and embryonic Polycomb and heterochromatin regulator dosage is required during distinct paternal and embryonic windows to mediate this intergenerational control in wild-type animals. This study identified 5 of the first known genes absolutely required for proper intergenerational metabolic reprogramming (IGMR) [126]. Intriguingly, the same machinery seems to modulate obesity susceptibility in isogenic mouse and human cohorts.

Exploiting the same principle in mice, the Whitelaw lab performed a large-scale mutagenesis screen for epigenetic regulators. Analogous to transgenic variegating *white* eye-color reporters, the Whitelaw group used an α-globin promoter driven GFP transgene array expressing in red blood cells as the foundation of an F2 mutagenesis screen. In mice, multicopy transgene arrays are particularly sensitive to repeat-associated gene silencing and often display variegation [128]. Screening more than 5000 G_1_ offspring for changes in GFP expression pattern the group identified more than 40 strains with either enhanced or reduced GFP expression as the result of heritable, dominant-acting mutations, referred to as *Momme*'s: Modifiers of Murine Metastable Epialleles [129], [130]. Although a significant overlap between *Momme* mutations in mice and Su(var) and E(var) in *Drosophila* exists, several components in the mouse do not have orthologues in the fly. Thus extra layers of regulation arose in mammalian cells, DNA methylation being a notable example [131]. Our own recent observations of Momme and related alleles include examinations of two lines originally reported to exhibit phenotypic noise [132], and, in particular, MommeD9, a point mutation in the KRAB-zinc-finger transcription factor TRIM28 (*Trim28*^*+/D9*^). This gene was identified as being an E(var) [132]. Re-examining those animals, we find that *Trim28*^*+/D9*^ animals exhibit a bimodal distribution in their body weight, emerging into adulthood as either normal or overweight, with few intermediates [142]. At the molecular level, the obese population displays specific dysregulation of a cluster of maternally imprinted or paternally expressed genes. Mutation of several of these ‘PEGs’, and in particular *Nnat*, is sufficient to independently recapitulate the phenotype. Given the stability of the distinct phenotypes, the non-Mendelian nature of heritability of the phenotype, and the highly inbred nature of the lines examined to date, these strains provide precedent for polyphenism in mice. They also identify a network of genes that buffer against dramatic ‘on-off’ like variation in mammals. Interestingly, analogous *Trim28*-associated transcriptomic dysregulation can also be observed specifically in *Trim28*-low expressing human children [127]. Thus, *Trim28*, *Nnat*, and the related PEGs represent molecular mediators of canalization itself and the focusing of developmental trajectories. A comparably unusual example highlighting stark non-Mendelian phenotypic variation are intergenerational developmental impairments caused by parental insufficiency in an enzyme of the folate pathway, *Mtrr* [133]. These clear examples of inducible multi-stable or stochastic variation highlight how little we know about the landscape of potential phenotypic variation itself.

Cellular systems for examining variegation-like phenotypes have now been developed. These have identified novel epigenetic regulators such as the HUSH H3K9me3-silencing complex and could serve as important tools for dissecting chromatin-environment regulatory axes [134].

#### 3.2.2 A role for true stochasticity, randomness, and noise?

Studying highly inbred lines of rodents, extensive phenotypic variation in diverse traits has been reported, even when they are grown in controlled environments [135]. In a study of outbred control population, 42% of the phenotypic variation in piebald pigment pattern was attributed to genes, the contribution of the environment was very small and the remaining >50% could not be attributed to any known process and was therefore considered the result of “the intangible sort of causes to which the word chance is applied” [136]. Another way in which this effect has been described is as a “third component”, additional with respect to genes and environment [135]. These phenomena and many monozygotic twin discordances have been attributed to stochastic sources of variation [139], variation that has been described as affecting all major evolutionary lineages, affecting morphological traits, biochemical and physiological traits, reproduction, life span, mortality as well as behavioral traits [140].

Stochastic or random drivers of variation can be genetic. Random Transposable Elements (TE) mobilization in specific tissues can cause deletions, inversions, translocations, or duplications. These events can then generate variation in gene expression, thus new phenotypes. This is not an irrelevant eventuality: around 400 million retrotransposon-derived structural variants are present in the human population, with at least 50% of the human genome derived from these sequences [141] and more than 70 diseases involving heritable and *de novo* retrotransposition events [142]. Somatic mosaicism driven by retrotransposition has been demonstrated to occur in the brain during neurogenesis, when LINE1 promoter is transiently released from epigenetic suppression [143], thus reshaping the genetic circuitry that underpins neurobiological processes [144].

Further, stochastic variation can be non-genetic affecting macromolecules, kinetics of reactions, cell-to-cell contact, mechanical force transduction, protein–protein promiscuity or catalysis, aggregation, and stochastic partitioning of cell components between daughter cells. While most processes in the cell or body are susceptible to generating random variation [145], as they rely, to some degree, on stochastic molecular interactions, these processes tend to be buffered or canalized. Thus, stochastic variation is, to some extent, the result of loss-of or imperfect buffering. Inherent stochastic variation has been implicated in biological processes at least since the 50s [146]. Regulatory pathways often take advantage of bistable loops in which molecular noise can be exploited to generate a signal. Furthermore, it has been proposed that stochasticity can provide a mechanism for phenotypic and cell-type diversification, allowing cells to “sample” distinct physiological states in order to increase the chance of survival in case of environmental change. Indeed, diversification can be the key to avoid being trapped in sub-optimal phenotypic states [145]. Transcription exhibits stochasticity, in its bursting activity, and involves interaction between transcription factors and RNA polymerase through partially random encounters [147]. While yeast promoter evolution is influenced by a need to maintain low levels of expression noise, acting through purifying selection, the situation is more complex in animals [148]. Noisy alleles are quite common within natural populations but appear to be buffered by allelic interactions with other variants that attenuate the effect [148]. Fluorescence microscopy indicates that the concentration of specific proteins in population of genetically identical cells differs from cell to cell on the order of tens of percent of the mean [149]. The broad variation of fingerprints in humans is thought to depends to a large degree on stochastic variation in mechanical forces [150]. Stochasticity has been proposed to have a great impact in disease susceptibility, as stem cell proliferation in adult tissue includes stochastic components and fluctuations in intercellular signaling seem to be involved in the stimulation of cancer stem cell development [151].

The overall robustness of the mechanisms that buffer against control of stochastic variation is highlighted by the relative identity of most monozygotic co–twin pairs. Noise control is mediated by feedback loops, redundancy, modularity, clustering, as well as by specific molecules. Intriguingly, this variation is also dependent on genetic background, that serves as the template for both genetic and epigenetic control systems. Indeed, mutations can remain silent until their unmasking for instance by stress. This is the case of mutations in sequences of *cis*-regulatory DNA and proteins regulated by Hsp90, a chaperone protein. Neutral as well as deleterious mutations are tolerated as far as Hsp90 can efficiently perform its function. In case of challenging environment, such as a rise in temperature, Hsp90 can be titrated away for other needs so that it cannot perform its buffering function anymore, allowing cryptic mutations to express their detrimental or beneficial effects [152]. Recently Hsp90 was shown to exert another role in uncoupling phenotypic outcomes from individual genotypes. Hsp90 indeed buffers *cis*-regulatory variation repressing the regulatory impact of endogenous retroviruses (ERVs) on neighboring genes. This function is critical for mouse development and is exerted thanks to a TRIM28-mediated epigenetic pathway [153]. Hsp90 has such a high impact that this effect induces phenotypic variation affecting nearly any adult structure in *Drosophila* [152] as well as a fifth of all the variation in the yeast genome [154]. For further reading regarding this broad topic, we refer to an extremely interesting and detailed review [140].

### 3.3 Conclusions

Though we know that some periods are more important that others, the field is still actively looking for a better definition of the most significant windows of sensitivity as well as the most impacting environmental stimuli. Adulthood retains some plasticity. Importantly, this residual plasticity has disease relevance. Perhaps the biggest unknowns beyond mechanism at the moment are a clear appreciation for the scope of impact of non-Mendelian variation on behavioural characteristics.

## 4 General conclusion

Phenotypic plasticity represents a measurable parameter that can be formulated as a statistical measure of variation or variance that can be partitioned as follows:

V_P_ = V_G_ + V_E_ + V_GxE_ + V_e_

This formula elegantly synthesizes what we expressed in the previous paragraphs: the total phenotypic variance of a trait is the sum of the genetic (Mendelian; V_G_) and the environmental variance (non-Mendelian; V_E_), the gene–environment interactions (V_GXE_) and other less defined sources of variation (stochastic; V_e_). Virtually any trait can show phenotypic plasticity, like morphology, biochemical and biological pathways, physiology, behavior, and life history that can be either reversible or irreversible throughout lifetime or even across generations [156]. Given the high interconnection among different traits, phenotypic variation often affects many different traits at the same time. A key question is whether such traits are primarily under genetic, non-Mendelian, or environmental control. The proportion of phenotypic variation attributable to genotypic variation is usually defined as ‘heritability’. Twin studies are the elective reference for this kind of studies, particularly when comparing monozygotic (MZ) and dizygotic (DZ) twins. Indeed, though they both share *in utero* environment, monozygotic twins are more genetically similar. Recent large studies of European twins, for example, have estimated that the heritability of stature is around 80–90% [157], [158]. GWA studies identified many common genetic variants for adult height that together explain around 56% of the variance [159]. BMI and waist circumference show slightly lower heritability values (∼77%) in a systematic review of twin studies [160], and consensus estimates of heritability for obesity and T2D are ∼70% and ∼35% respectively [161], [162]. The remaining, unexplained component is known to involve gene–environment interactions as well as non-Mendelian players. The current outstanding challenge remains mechanistically dissecting the latter and their contribution to human affliction, agriculture, ecological robustness and so on.

## 5 Outlook

An improved understanding of fundamental mechanisms driving the emergence of complex traits in otherwise isogenic individuals will set the basis for a deeper comprehension of differential disease susceptibility and thus underpin diagnostic and therapeutic advance. The ultimate goal of personalized medicine is indeed to identify genetic variants and epi-mutations associated with common diseases to forecast disease susceptibility and optimize treatment to the exact nature of an individual's disease state. Two obstacles block this path: first, the complexity behind the ‘genotype – phenotype’ relationship and, second, our ability to predict exact outcome and not ‘typical’ or average outcomes of a given scenario. Lack of detailed information on the maternal and fetal environments throughout gestation and of longitudinal studies in humans limit our understanding of the impact of prenatal insults on adult development of metabolic diseases. The identification of the players that mediate epigenetic inheritance will help us understand the landscape of altered personal phenotypic potential. Given the relationship between epigenetic machinery and cellular metabolism metabolites, nutrition and the microbiome provide opportunities for intervention and reversal of non-Mendelian effects. So, while the challenges remain great, there is a clear way forward. Regardless of the complexities, the pursuit to further understand disease “epi-types” represents a worthwhile endeavor and will improve our rates of successfully improving human health.

Given the biases against publication of negative and/or contradictory data, a continued challenge for the field is a clear evaluation of the reproducibility, robustness and variability of plasticity effects, as well as clear documentation of interventions that have *no effect*. Currently, the field suffers from a lack of standard operating procedures and efforts to evaluate robustness of the myriad models that exist. Indeed, since phenotypic variation is affected by so many environmental variables (paternal and maternal stress and environmental exposures, litter size, lactation sufficiency, maternal care, etc.), effective experimental control is a major barrier to proper investigation. Standards for experimental design and animal numbers are needed, as is double-blinded validation, ideally by independent labs and prior to publication.

While intertwined with funding and competition issues, these barriers are not trivial. That said, overcoming them will be worth it. Each and every individual has his or her own unique genetic background, epigenetic overlay, parental and ancestral history, and environment. Dissecting the most potent epigenetic regulatory layers governing phenotypic variation should be a high goal. It is one of the key gateways to personalized medicine and will improve health outcomes for all disease.
